# Identifying risk factors affecting exercise behavior among overweight or obese individuals in China

**DOI:** 10.3389/fpubh.2023.1122473

**Published:** 2023-06-22

**Authors:** Guo Shengyu, Feiyue Liu, Qinghua Wu

**Affiliations:** Department of Economics and Management, Changsha University, Changsha, China

**Keywords:** obesity, exercise behavior, risk factors, overweight, self-rated health

## Abstract

**Background:**

The disease burden caused by obesity has increased significantly in China. Less than 30% of those who are obese meet the weekly physical activity standards recommended by the WHO. Risk factors that influence exercise behavior in people with obesity remain unclear.

**Methods:**

Based on the survey from the Chinese General Social Survey program (CGSS) in 2017, 3,331 subjects were identified and enrolled in the univariate and multiple probit regression models. We aimed to identify the association between SRH and the exercise behavior of obese people and further explore the influencing factors of active physical activity in this group of people.

**Results:**

The proportion of active physical activity in obese people was 25%. Groups with better SRH, higher education and income were more likely to participate in sports. Obese people who lived in rural areas, were unmarried or divorced, or fell within the age range of 35–40 had a significantly lower percentage of engagement in active physical activity.

**Conclusions:**

The proportion of people with obesity who meet the WHO recommendation for physical activity in China is not ideal. Health promotion programs for those who are obese need to be further strengthened and targeted, especially for rural areas, low-income families, and middle-aged obese people.

## Introduction

Obesity has become an important public health problem around the world and has a significant impact on quality of life (1). Many scholars have conducted studies on the side effects of obesity, and increasing evidence has confirmed that obesity is significantly associated with the occurrence of some diseases, including hypertension (2), diabetes (3), coronary heart disease (CHD) (4), cancer (5), and stroke (6). According to a retrospective study based on a large number of samples in Britain, obesity may be an important risk factor for premature death (7).

Chinese scholars have also conducted studies that focused on several aspects. First, they described the distribution characteristics of people who are overweight and obese in the Chinese population and also analyzed the changing trends in the burden of being overweight and obese in the country (8, 9). Second, these researchers explored the influencing factors and related mechanisms of obesity through animal experiments and population surveys (10, 11). Another important research direction was to explore how to diminish the incidence of obesity and reduce the burden of obesity-related diseases based on the previous two aspects (12). Although these studies have played an important role in controlling obesity in China, the disease burden of obesity in China has been showing a rapid growth trend. It is estimated that more than half of the adults in China are overweight or obese, which has become one of the most serious public health concerns in this country (13). Thus, reducing the disease burden caused by obesity has become a major problem affecting the realization of the Healthy China 2030 strategy.

The World Health Organization (WHO) has launched a guideline to prevent and control obesity. The most effective measures to reduce the disease burden of obesity are non-pharmaceutical interventions (NPI), including dietary intervention (reducing nutrient intake) and exercise intervention (increasing fat reduction). For the latter, evidence-based recommendations are provided on the volume of exercise needed to keep fit. For adults, for example, they should get at least 150–300 min of moderate-intensity aerobic exercise per week or participate in no <75–150 min of high-intensity aerobic activity. Moreover, considering the effects of exercise on the health of humans, the population was divided into four groups: (1) inactive group, who have no aerobic physical activity or who seldom take part in exercise; (2) insufficiently active group, who are involved in some aerobic activity each week but less than the aerobic guideline (150–300 min of moderate-intensity exercise); (3) active group (AG), who meet the guideline of participating in aerobic exercise for 300 min per week; (4) sufficiently active group (SAG), who exceed the aerobic guideline of >300 min per week.

The Chinese local government has launched a series of health policies and actions to promote active physical activity (14). However, such policies and actions are not followed by all adults. Previous investigations by our team have found that the proportion of people actively participating in sports every week is not ideal. Some factors, such as culture (15), environment (16), family (17), and community facilities (18), may have a significant impact on the exercise behavior of residents. The recognition of obesity may also be an important risk factor. The Chinese have traditionally viewed obesity as a symbol of wealth. Many of them do not consider obesity an unhealthy condition or disease. In addition, there is not a significant impact on the quality of life in the early stages of being overweight or obese, and the self-rated health of obese individuals may also be a factor that motivates them to participate actively in sports.

**Figure 1:**
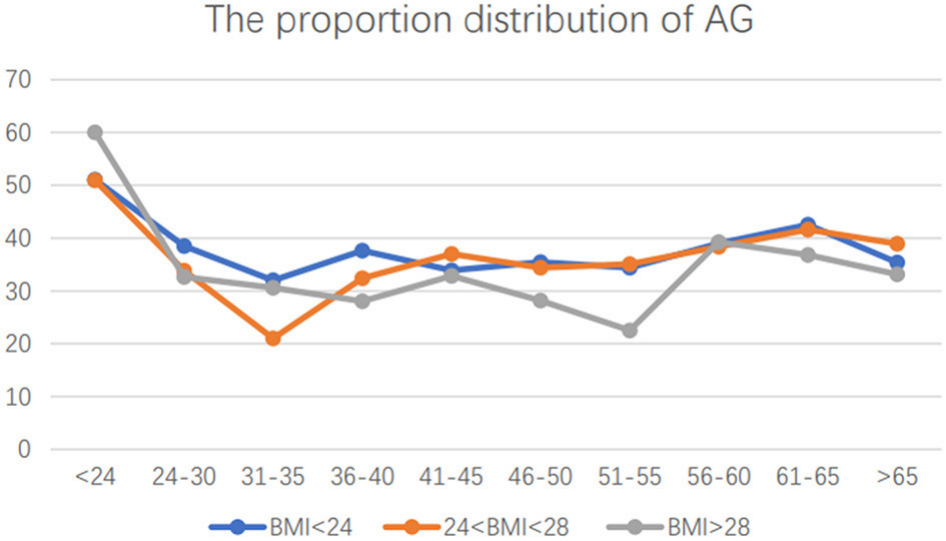
The proportion of AG in overweight, obese, and normal-weight samples.

The Chinese General Social Survey (CGSS) program was a nationally representative study (19). With data from CGSS 2017, we first compared the exercise behavior of obese individuals and normal-weight groups, and there was no significant difference between the two groups (detailed results are shown in Table 1, Figure 1).

**Table 1:** Distribution characteristics of exercise behavior of people with different ages and BMI index.

**Age**	**BMI**<**24**		**24**<**BMI**<**28**		**BMI** > **28**		**χ^2^**	***p*-value**
	***n***	***n*** **.sport**	***n***	***n*** **.sport**	***n***	***n*** **.sport**		
All	5,668	2,109 (37.21%)	2,543	931 (36.61%)	774	257 (33.20%)	9.75	0.007^**^
<24	276	141 (51.08)	104	53 (50.96)	30	18 (60.0)	0.89	0.64
24–30	400	154 (38.5)	192	65 (33.85)	49	16 (32.65)	1.57	0.45
31–35	447	143 (32.0)	176	37 (21.02)	49	15 (30.61)	6.66	0.03^*^
36–40	425	160 (37.65)	185	60 (32.43)	57	16 (28.07)	4.04	0.13
41–45	437	148 (33.87)	181	67 (37.01)	64	21 (32.81)	0.66	0.71
46–50	567	201 (35.45)	250	86 (34.4)	78	22 (28.20)	1.59	0.45
51–55	622	214 (34.40)	262	92 (35.11)	71	16 (22.53)	4.33	0.11
56–60	518	202 (39.0)	237	91 (38.39)	79	31 (39.24)	0.03	0.98
61–65	646	275 (42.57)	286	119 (41.60)	95	35 (36.84)	1.12	0.57
>65	1,330	471 (35.41)	670	261 (38.95)	202	67 (33.16)	7.52	0.02^*^

^*^means *p* < 0.05, ^**^means *p* < 0.001.

Therefore, our attention was drawn to a question: Although exercise is the best way to stay healthy, why do people with obesity not like to take part in it?

Moreover, previous studies on exercise behavior among obese individuals have mainly focused on the effects of exercise on weight loss (20–23). However, limited studies have focused on the distribution of exercise behavior in overweight and obese individuals, and the risk factors of regular exercise among this special group remain unclear. Therefore, this situation constitutes a significant gap between action assessment and available data, which may affect the precise regulation of health promotion policies.

The main purpose of this study is to describe the distribution characteristics of exercise behavior and explore the factors that affect the exercise behavior of this group. Thus, we can provide guidance to policymakers and other researchers in identifying the important problems of obesity intervention in China at the current stage and enacting proactive measures to promote the exercise participation behavior of obese people.

We also hypothesized that self-rated health might influence the exercise behavior of those who are obese. By verifying the hypothesis and exploring the influencing factors of the exercise behavior of those who are obese, we intended to provide a scientific basis for the improvement of obesity intervention strategies in China.

## Methods

The data for this study were derived from the program CGSS2017, which is a nationally representative study containing a multi-stage random sampling across the country. The detailed study design and procedures of this program were previously reported in the original study documentation (19). Data from CGSS2017 were first made public in 2020, and 8,999 samples were included in the program. All data were downloaded publicly (http://cgss.ruc.edu.cn/) in January 2021. Samples were excluded based on the following criteria: (1) Some quantitative variables, such as family income and age, were not answered accurately, such as an income of 0 and an age of more than 120; (2) missing values >5% for family factors and other important variables were excluded from the study; and (3) according to the height and weight of each sample, we first calculated the BMI of each sample, and this study only included the samples whose BMI was >24.

### Independent variable

In this study, the quantitative evaluation of sports among those who are obese was considered the independent variable. A question about exercise behavior was asked in the survey: “In the past 12 months, you have engaged in physical activity that usually lasts up to 30 minutes and causes you to sweat. If yes, how many times a week?” Based on the guidelines of the WHO (adults should get at least 150–300 min of moderate-intensity aerobic exercise per week or participate in no <75–150 min of high-intensity aerobic activity), the included samples were divided into two groups: the active group and the inactive group (24).

### Dependent variable

In this study, self-rated health (SRH) is the variable that contains self-perceived physical health, self-perceived depression, and the impact of health on life. A question referred to self-perceived physical health: “How do you feel about your current physical health?” Options include “very unhealthy,” coded as 1, “less healthy,” coded as 2, “general,” coded as 3, “healthy,” coded as 4, and “very healthy,” coded as 5, respectively. The average score for this variable in different populations was assessed as a separate variable to evaluate the level of self-perceived physical health. Another item referred to the frequency of depression during the past 4 weeks (“How often have you felt depressed in the past 4 weeks?”). Responses to the question were coded as a quantity range from “1” to “5” (“always” = 1, “frequently” = 2, “sometimes” = 3, “seldom” = 4, or “never” = 5). The question “How often in the past 4 weeks have health problems affected your work or other daily activities?” referred to the impact of health on life. The score of this item was also 1–5. In this study, the SRH score ranged from 5 to 15, with higher scores indicating higher levels of SRH.

### Covariate variables

In this study, age was an important factor that was divided into 10 groups. We compared the exercise behaviors of different ages, hoping to provide precise guidance for health intervention.

BMI (body mass index), which is used to measure how fat a person is and whether they are healthy, was calculated using the following formula: BMI = weight (kg)/height^2^ (m).

The World Health Organization (WHO) defines being overweight as a BMI of >25 and obesity as a BMI of >30. However, the more common standard in China for being overweight was a BMI of >24, and for being obese, a BMI of >28 (25, 26).

Regarding family income, a question in the questionnaire asked: “What level do you think family income belongs to in the local area?” The answers were “1-below the average line,” “2-above the average line,” and “3-above the average line.”

Educational background categories were as follows: elementary school and below, middle school, high school, and college and above.

Owing to the significant development gap among different areas in China, all the included samples were divided into urban and rural groups according to the Hukou variable.

### Statistical analysis

Data analyses were performed using R software (3.5.2), and frequencies and percentages of factors were calculated for descriptive analysis. For numerical variables, bivariate analyses were performed using Pearson's chi-square test, and the association between variables and exercise was assessed using univariate and multinomial logistic regression models. Binary logistic regression analysis with the “enter” method was first used to examine dependent correlates of exercise, with exercise as a dependent variable and those with significant differences in univariate analyses as independent variables. The association between variables and exercise behavior was described with an odds ratio (OR) and 95% confidence interval (CI). The level of significance was set at a *p*-value of <0.05 (two-tailed *t*-test).

## Results

### Descriptive analysis

A total of 8,999 random national samples were included in the CGSS 2017 program, and according to the inclusion and exclusion criteria, 3,331 samples (2,543 overweight and 788 obese) with high BMIs were included in this study. Overall, the proportion of overweight people meeting the recommended physical activity standards of the WHO was lower than that of people of healthy weight. The compliance rates of the healthy population, overweight group, and obesity group are shown in Table 1.

To explore the differences in physical activity between those who were overweight or obese and the general population, we stratified the samples by age and subdivided them into 10 groups. Table 1 shows the proportion of those who meet the WHO recommendation on physical activity. Ideally, those who are overweight or obese should have higher rates of physical activity than the general population. However, the results showed that the proportion of participation in sports was significantly different only in two age groups (31–35 and >65). Moreover, it is particularly important to note that the proportion of obese people was significantly lower than that of normal-weight people at the age of 31–35, which indicates that the low proportion of regular exercise among those who are overweight or obese in China is an important public health problem (Figure 1). The basic descriptive statistics of those who are overweight or obese are shown in Table 2. Among those who are overweight or obese, the proportion of women who meet the exercise standard was higher than that of men (women: 39.02%, men:32.0%), the proportion of people in the married group (34.1%) was higher than that of the those in the divorced group (28.9%), and the proportion of urban residents (42.45%) was higher than that of rural residents (21.88%).

**Table 2:** Demographic and sociological characteristics and univariate analysis of exercise behavior of overweight people.

**Variables**	**Group**	***n***	***n*.sport**	**%**	***χ^2^***	***p*-value**
Gender	Male	1,586	507	32.0	20.32	6.528e-06
	Female	1,745	681	39.02		
Age	<24	134	71	52.98	212.18	<2.2e-16
	24–30	241	81	33.60		
	31–35	239	62	25.94		
	36–40	242	76	31.40		
	41–45	245	88	35.92		
	46–50	328	108	32.92		
	51–55	333	108	32.43		
	56–60	316	122	38.60		
	61–65	281	154	54.80		
	>65	872	328	37.61		
BMI	24–28	2,543	931	36.61	1.82	0.17
	>28	788	267	33.88		
Physical health	Good	1,878	756	40.25	67.59	2.10e-15
	General	855	302	35.32		
	Bad	598	140	23.41		
Mental health	Good	2,264	795	36.84	3.76	0.04
	Bad	1,067	409	38.33		
Impact of health on life	Seldom	1,406	258	18.34	55.43	9.196e-13
	Sometimes	969	350	36.11		
	Frequent	956	590	61.71		
Education	1	984	237	24.08	142.77	<2.2e-16
	2	1,000	329	32.90		
	3	627	269	42.90		
	4	720	363	50.41		
Marriage	Divorced	730	211	28.90	6.73	0.01
	Married	2,601	887	34.10		
Income	Subaverage	723	71	9.82	277.3	<2.2e-16
	Average	2,437	1,064	43.66		
	Above average	171	63	36.84		
Hukou	City	2,280	968	42.45	131.2	
	Rural	1,051	230	21.88		

^*^means *p* < 0.05, ^**^means *p* < 0.001, ^***^means *p* < 0.0001.

### Univariate analysis

The results of the univariate analysis indicated that obese men are less active than women, and significant differences existed in exercise participation among obese people of different ages. Overweight individuals in two age groups (<24 and 60–65 years) had a higher proportion of exercise participation, and the proportion of exercise reaching the standard (amount of exercise) was more than 50%. The minimum proportion of exercise that reached the standard was in the 30–35 age group (25.94%). There was no significant difference in exercise behavior between the overweight and obese groups (36.61 and 33.88%), but a significant difference in exercise behavior was found between the urban and rural groups (42.45 and 21.88%). Educational background also had a significant impact on the exercise behavior of obese people, and the result of the analysis showed that education level was positively correlated with active participation in sports. The samples with a college education or above had the highest proportion of exercise participation behavior (50.41%). Additionally, the three dimensions of self-rated health had a significant relationship with regular exercise (*p* < 0.05). The results of the univariate analysis also showed that sex (*p* < 0.00), family income (*p* < 0.00), hukou (*p* < 0.00), and marital status (*p* = 0.01) were also significantly related to the exercise behavior of those who are overweight. Detailed results are shown in Table 2.

### Multivariate analysis

To identify the association between self-rated health and exercise behavior, variables that were significant in the univariate analysis were further incorporated into the multivariate models, and the results indicated that better physical health (OR = 1.14, 95% CI: 1.03–1.19) might significantly improve the exercise behavior of those who are overweight or obese, while poor mental health may reduce the likelihood of participation in sports (OR = 0.95, 95% CI: 0.93–0.98). As shown in Table 3, other variables, such as education, family income, hukou, and marital status, were also significantly related to the exercise behavior of obese people in the multivariate model.

**Table 3:** Multivariate analysis of exercise behavior in obese people.

**Variables**	***B***	**SD**	***Wald χ^2^***	***p*-value**	**OR**	**95%CI**	
						**Lower**	**Upper**
**Hukou (Ref** = **city)**							
Rural	−0.52	0.04	−12.99	<2e−16 ^***^	0.59	0.54	0.64
**Gender (ref** = **male)**							
Female	−0.05	0.03	−1.28	0.19	0.95	0.88	1.02
	−0.07	0.03	−1.56	0.11	0.93	0.85	1.01
**Age (ref** = <**24)**							
24–30	−0.26	0.11	−2.36	0.01^*^	0.76	0.61	0.95
31–35	−0.38	0.11	−3.39	0.0006^***^	0.68	0.54	0.85
36–40	−0.30	0.11	−2.67	0.007^**^	0.73	0.59	0.92
41–45	−0.21	0.11	−1.86	0.06	0.80	0.64	1.01
46–50	−0.24	0.10	−2.25	0.02^*^	0.78	0.63	0.96
51–55	−0.21	0.10	−1.94	0.052	0.81	0.65	1.00
56–60	−0.06	0.10	−0.62	0.53	0.93	0.75	1.15
61–65	0.08	0.10	0.76	0.44	1.08	0.88	1.34
>65	0.04	0.10	0.41	0.68	1.04	0.85	1.26
**Physical health (ref** = **bad)**							
General	0.04	0.02	1.69	0.09	1.04	0.99	1.09
Good	0.13	0.02	4.85	0.0001^***^	1.14	1.03	1.19
**Self-health (ref** = **frequent)**							
Sometimes	0.02	0.02	1.04	0.29	1.02	0.97	1.04
Seldom	0.03	0.02	1.45	0.14	1.03	0.98	1.06
**Mental health (ref** = **health)**							
Unhealth	−0.04	0.01	−2.91	0.003^**^	0.95	0.93	0.98
**Income (ref** = **below)**							
General	0.12	0.01	7.27	<0.000^***^	1.13	1.09	1.17
Above	0.22	0.03	6.90	<0.000^***^	1.25	1.17	1.34
**Education (ref** = **1)**							
2	0.04	0.01	2.73	<0.000^***^	1.04	1.01	1.08
3	0.07	0.01	3.87	<0.000^***^	1.07	1.03	1.11
4	0.12	0.01	6.54	<0.000^***^	1.13	1.08	1.17
**Marriage (ref** = **married)**							
Divorced	−0.04	0.01	−3.00	0.002^**^	0.95	0.92	0.98

^*^ <0.05.

^**^ <0.001.

^***^ <0.0001.

## Discussion

Obesity has become an important challenge that needs an urgent response in China. The WHO has recommended exercise guidelines for different groups. However, many of them do not follow the guidelines. Based on the data from the CGSS 2017 program, we sought to explore the related factors that affect the exercise behavior of obese people, especially the influence of self-perceived health (SRH) on exercise.

### Exercise behavior in overweight and obese people needs to be promoted

One major finding of this study was that the proportion of AG in overweight and obese people was not ideal and even lower than in people of healthy weight. Previous studies have explained the reasons for this phenomenon mainly from two angles: individual factors and environmental factors (27, 28). The former holds that factors such as personal health literacy and cognition of obesity will have an important impact on exercise participation behavior. Better health literacy helps them to participate more in sports, and those who were aware of the serious medical consequences of obesity were more likely to engage in exercise (29). At present, the Healthy China 2030 strategy is being vigorously promoted, and one of its main purposes is to improve people's health literacy and promote healthy behavior (30, 31).

This study found that age was another important individual factor. The exercise behavior of those who are obese in middle age should be given special attention, especially in the 31–40 and 46–50 age groups. The health status of middle-aged people is attracting great attention from scholars and public health experts. In the past, it was believed that the health status of this population was better than other groups, but more and more studies have shown that the peak incidence of some noncommunicable diseases (NCDS) is shifting to the population (32, 33). The rate of sudden death at this age is also increasing rapidly (34, 35). There may be two possible reasons: first, the traditional view is that this group is the backbone of society, subject to more work and pressure, and has less time and opportunity to exercise or relax. Second, compared with older people (>60), this group does not feel physically uncomfortable and has a good level of SRH in the early stages of some NCDS, which will also affect their health behaviors.

### SRH is significantly associated with the health behaviors of those who are overweight or obese

In this study, SRH was defined as a subjective evaluation of health based on which people may adopt different health behaviors (36, 37). Whether SRH has any effect on health behaviors, such as exercise among those who are obese, remains unclear. Previous studies have proven that obesity is a statistically significant predictor of reduced SRH. A similar conclusion was observed not only in adults but also in children and adolescents (38). In China, obesity is traditionally regarded as a sign of wealth or beauty, which may affect the correct perception of obesity. Moreover, the results of this study showed that SRH did have a significant effect on the exercise behavior of obese individuals; those with a better SRH are more likely to participate in sports. Meyer et al. (39) found that SRH is critical for tailoring interventions and designing programs that can promote physical activity, which is consistent with the conclusion of our study.

From the perspective of public health, reversing the rapid increase in obesity among young and middle-aged people is also an important measure to reduce the disease burden caused by obesity in China.

### Environmental factors have a significant influence on the health behavior of those who are overweight or obese

Consistent with some important research (40, 41), this study found that environmental factors such as income, educational background, residential community, and marriage have an important impact on physical activity. Poor income levels and lower education are negatively associated with physical activity, which was highly consistent with the results of this study. The underlying mechanism may be that higher educational background and income are associated with greater social capital, which significantly improves the health literacy of humans (42).

In China, hukou is an important variable that divides urban and rural residents into different communities, and we found that the proportion of those who engage in regular exercise in rural areas was significantly lower than that of urban residents. Rural areas have witnessed rapid development in the past decades, and the food supply for the masses is abundant. However, another problem is that the rate of obesity has also increased significantly, and the increase is greater than the overall population. In recent years, China has promoted the rural revitalization strategy to reduce the huge gap between urban and rural areas. The gap between urban and rural areas includes not only the economic gap but also the gap in health literacy and health behavior of urban and rural residents (43). Only by reducing the health gap between urban and rural residents can rural revitalization be truly realized. Thus, this study can provide important guidance for improving public health services in rural areas.

## Limitations

Several major limitations of this study should be noted. First, although the samples in this study are fairly representative, and the latest data are available, the data in this study reflect the situation in 2017, and the conclusions may be different from the current situation in China. Second, this is a cross-sectional study, so any conclusions are only understood as statistically significant but do not provide conclusive causality. Another limitation was that this was an observational study based on public data, and owing to the absence of specific variables such as lifestyle, we could not fully understand the risk factors affecting exercise behavior among those who were obese. Therefore, future studies need to gather more variables in this area so that higher-value scientific evidence can be provided to reduce the disease burden of obesity.

## Conclusions

In summary, there is a high incidence of obesity in China, and only <30% of the population meets the recommended guidelines of the WHO regarding the amount of physical activity. Among those who are overweight, those aged 31–35 years were the least physically active, and the proportion of rural residents participating in exercise was significantly lower than that of urban residents. Lower SRH scores, family income, divorce, and educational background significantly negatively affected exercise behavior in obese people.

### Suggestions

Interventions should be developed to help those who are overweight or obese based on the findings of this study.

First, health education on obesity should be improved. The proportion of overweight and obese people who reached the target of physical activity was lower than that of normal-weight people, which indicated that many of them do not correctly understand the important impact of obesity on health. According to the theory of planned behavior (TBP), only by changing the wrong perception of obesity can their exercise behavior be increased. Therefore, the existing contents of health education need to be incorporated to emphasize the understanding that “obesity is a disease.” Moreover, it is also worth advocating online health education. More sports guidance videos should be launched on APPs, TikTok, Twitter, and other platforms.

Second, the health literacy of rural residents needs to be further improved. There is a significant difference in the proportion of obese people participating in exercise between urban and rural areas. Based on the analysis of relevant big data, more targeted health promotion services should be provided to rural residents.

Finally, more sports spaces and facilities should be built for communities. For example, sports venues in many Chinese cities are commercialized and open only to those who can afford them, which may limit the enthusiasm of other groups. Community sports resources should be co-managed to help all residents accept and use them by providing exercise guidelines and financial subsidies (reducing the cost of participation).

## Data availability statement

The original contributions presented in the study are included in the article/supplementary material, further inquiries can be directed to the corresponding author.

## Author contributions

GS: conceptualization, methodology, and writing. FL and QW: data analysis. All authors contributed to the article and approved the submitted version.
